# Chemical shift assignments of the N-terminal domain of PSD95 (PSD95-NT)

**DOI:** 10.1007/s12104-021-10028-5

**Published:** 2021-04-30

**Authors:** Yonghong Zhang, Johannes W. Hell, James B. Ames

**Affiliations:** 1 Department of Chemistry, University of California, Davis, CA 95616 USA; 2 Department of Pharmacology, University of California, Davis, CA 95616 USA; 3 Present Address: Department of Chemistry, The University of Texas Rio Grande Valley, Edinburg, TX 78539 USA

**Keywords:** PSD95, Calcium, Glutamate receptor, Postsynaptic density, NMR

## Abstract

Postsynaptic density protein-95 (PSD95) contributes to the postsynaptic architecture of neuronal synapses and plays an important role in controlling synaptic plasticity. The N-terminal domain of PSD95 (residues 1–71, called PSD95-NT) interacts with target proteins (calmodulin, α-actinin-1 and CDKL5), which regulate the Ca^2+^-dependent degradation of glutamate receptors. We report complete backbone NMR chemical shift assignments of PSD95-NT (BMRB No. 50752).

## Biological context

PSD95 is a membrane-associated protein composed of three PDZ domains, followed by a src homology 3 (SH3) domain and a C-terminal guanylate kinase region. The N-terminal region of PSD95 (residues 1–71) interacts with a number of target proteins, such as calmodulin (Zhang et al. 2014), α-actinin-1 (Matt et al. 2018), and CDKL5 (Zhu et al. 2013). PSD95-NT also contains palmitoylation sites that are important for Ca^2+^-dependent membrane targeting (Greaves et al. 2011). The PDZ domains in the N-terminal portion of PSD95 interact with specific (Ser/Thr)-Xxx-Val motifs at the C termini of the target proteins. Such interactions mediate PSD95 binding to the central GluN2 subunits of NMDA-type glutamate receptors and to the auxiliary stargazin and related TARP subunits of AMPA-type glutamate receptors (Lim et al. 2002; Xu 2011). Thus, PSD95 mediates postsynaptic targeting of these receptors (Elias et al. 2008; Schnell et al. 2002). PSD95 plays a central role in Ca^2+^-mediated regulation of postsynaptic glutamate receptor localization that occurs during long-term potentiation (LTP), which depends on Ca^2+^ influx through the NMDA receptor (Tomita et al. 2005; Sumioka et al. 2010). Furthermore, PSD95 is palmitoylated at N-terminal residues (Cys3 and Cys5), which is important for its postsynaptic localization and for postsynaptic targeting of AMPA receptors (El-Husseini et al. 2002). Intriguingly, Ca^2+^ influx also causes PSD95 relocalization (Sturgill et al. 2009; Zhang et al. 2014; Chowdhury et al. 2018).

Although structures are known for various PDZ domains and PSD95-NT bound to calmodulin (Zhang et al. 2014), atomic level structural information is currently not known for the full-length PSD95. We report here NMR resonance assignments of the N-terminal region of PSD95, which precedes the first PDZ domain and targets PSD95 (and bound glutamate receptors) to postsynaptic sites upon its palmitoylation of Cys3 and Cys5. These NMR assignments may be useful for the future screening and possible discovery of target proteins that bind to the PSD95 N-terminal domain.

## Methods and experiments

*Expression and purification of PSD95-NT.* A GST-tagged PSD95 N-terminal construct (residues, 1–71) (pGEX-4T3 vector) was transformed into *Escherichia coli* strain BL21(DE3) for protein overexpression and purification by following a standard protocol (Zhang et al. 2012). The ^15^ N/^13^C-labeled PSD95-NT was expressed in one liter of M9 medium supplemented with 0.5 g of ^15^ N-labeled NH_4_Cl and 2.5 g of ^13^C-labeled glucose. The purified GST-fusion protein was digested by thrombin to remove the N-terminal GST tag, producing an N-terminal fragment of PSD95 (residues 1–71, called PSD95-NT) that was further purified by gel-filtration size-exclusion chromatography (Superdex 75).

**Fig. 1:**
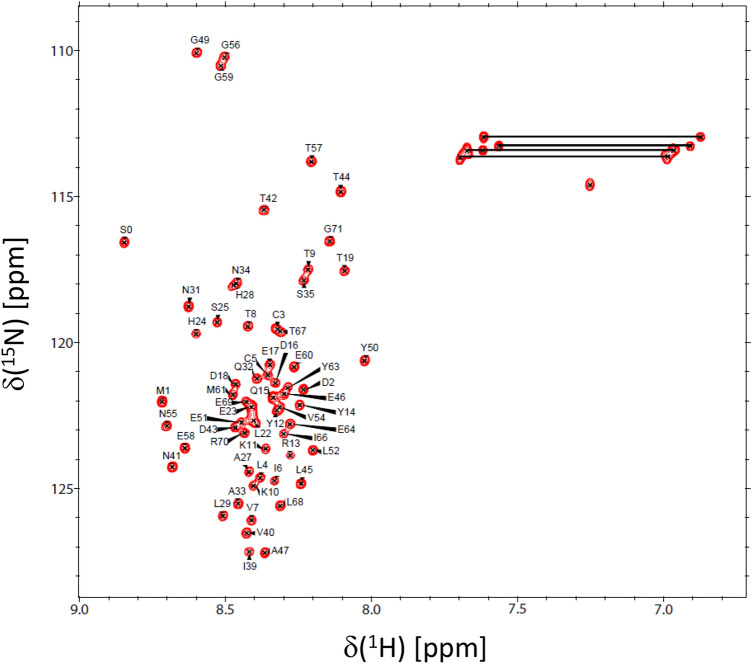
Two-dimensional ^15^ N-^1^H HSQC spectrum of ^15^ N-labeled PSD95(1–71) at pH 5.0 recorded at 800-MHz ^1^H frequency. Amide side-chain resonances are connected by solid lines. Resonance assignments are indicated and reported in BMRB accession no. 50752

*NMR spectroscopy.* Protein samples of ^15^ N- or ^13^C/^15^ N-labeled PSD95-NT were exchanged into NMR buffer containing 25 mM CD_3_COONa (pH 5.0) with 1 mM EDTA-d_12_, 1 mM DTT-*d*_*10*_ and 95% H_2_O/5% D_2_O. The isotopically labeled PSD95-NT was concentrated to give a final concentration of 0.4 mM in a final volume of 0.3 mL. All NMR experiments were performed at 285 K on a Bruker Avance III 800 MHz spectrometer equipped with a four-channel interface and triple resonance cryogenic (TCI) probe. The ^15^ N-^1^H HSQC spectrum (Fig. 1) was recorded with 256 × 2048 complex points for ^15^ N(F1) and ^1^H(F2). Assignment of backbone resonances was obtained by analyzing the following spectra: HNCACB, CBCA(CO)NH, HNCO and HBHA(CO)NH. The NMR data were processed using NMRPipe (Delaglio et al. 1995) and analyzed using Sparky (Lee et al. 2015).

### Assignments and data deposition

**Fig. 2:**
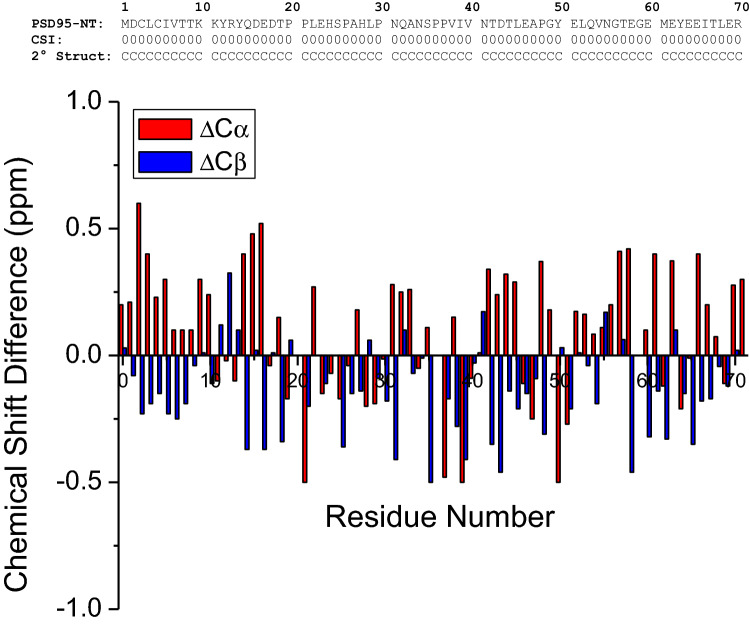
Primary and secondary structure of PSD95-NT. The secondary structure of each residue (random coil conformation depicted as C) was calculated on the basis of chemical shift index (CSI) as defined by (Wishart et al. 1992). The chemical shift difference (ΔCα and ΔCβ) for each residue was calculated as the observed chemical (for Cα and Cβ) minus the random coil chemical shift (Wishart et al. 1995) and is plotted as a function of residue number in the lower panel

Figure 1 presents the ^15^ N-^1^H HSQC spectrum of PSD95-NT to illustrate representative backbone resonance assignments. NMR assignments were based on 3D heteronuclear NMR experiments performed on ^13^C/^15^ N-labeled PSD95-NT. The resonances (^1^HN, ^15^ N, ^13^Cα, ^13^Cβ, ^1^Hα, ^1^Hβ, and ^13^CO) were detected and assigned for all 71 residues. A non-native serine residue was detected at the N-terminus due to a cloning artifact and is designated as S0. Surprisingly, the N-terminal serine residue exhibits an amide resonance in the HSQC spectrum, which suggests that the N-terminal amine group must be modified somehow as an amide, perhaps caused by N-acetyl modification or another N-acyl linkage at the N-terminus. The residue numbering begins with the methionine at the second position, designated as M1 (Figs. 1, 2). The amide proton chemical shifts exhibited very narrow chemical shift dispersion (within 8.0–8.7 ppm), suggesting a solvent exposed random coil conformation for the entire 71-residue peptide chain, which is consistent with a lack of any ring-current shifted amide or methyl resonances. The backbone chemical shift assignments (^1^H, ^15^ N, ^13^C) for PSD95-NT have been deposited in the BioMagResBank (http://www.bmrb.wisc.edu) under accession number 50752.

The previous NMR structure of calmodulin bound to PSD95-NT revealed a helical structure for the first 17 residues of PSD95 (Zhang et al. 2014). Thus, the binding of calmodulin sequesters the N-terminal residues, Cys3 and Cys5 and prevents their palmitoylation needed for membrane targeting. The secondary structure of PSD95-NT in the absence of calmodulin (calculated by chemical shift index (Wishart et al. 1992)) indicates a random coil conformation for the entire 71-residue peptide (Fig. 2) that we suggest may increase the exposure and availability of Cys3 and Cys5 for palmitoylation by palmitoyl transferase enzymes. The chemical shift differences (ΔCα and ΔCβ in Fig. 2) suggest a slight propensity for an α-helix in the first 10 residues from the N-terminus; however, the magnitude of the chemical shift differences here are quite small and less than 2-fold greater than the experimental error. The helical structure of the N-terminal residues of PSD95 (residues 1–17) bound to calmodulin must be stabilized by the binding of calmodulin, because the PSD95 N-terminal helix converts into a random coil in the absence of calmodulin. A similar structural change in PSD95-NT is likely to occur upon binding to other target proteins such as α-actinin-1 (Matt et al. 2018) and CDKL5 (Zhu et al. 2013; Zhang et al. 2014). The NMR chemical shifts reported here will be useful in the future for screening conformational changes in the N-terminal domain of PSD95 upon binding to the various protein targets.

## Data Availability

The assignments have been deposited to the BMRB under the accession code: 50,752.
